# Clinical review of adult Henoch–Schönlein purpura and analysis of predictors related to gastrointestinal involvement

**DOI:** 10.3389/fimmu.2025.1694532

**Published:** 2025-12-05

**Authors:** Ao Guo, Yujun Wang, Xiaojuan Nan, Yueshuai Zhang, Ping Liu, Chen Tu

**Affiliations:** Department of Dermatology, The Second Affiliated Hospital of Xi’an Jiaotong University, Xi’an, China

**Keywords:** Henoch-Schönlein purpura, IgA vasculitis, adults, gastrointestinal involvement, D-dimer

## Abstract

**Background:**

Henoch–Schönlein purpura (HSP) is a small-vessel vasculitis characterized by non-thrombocytopenic purpura, often involving the gastrointestinal tract, joints, kidneys, and other organs. While most studies on HSP focus on children, severe manifestations and poor outcomes are more frequently observed in adults.

**Objective:**

This retrospective study aimed to evaluate predictors associated with gastrointestinal (GI) involvement in adult patients.

**Methods:**

We included 206 adult patients with HSP, categorized into subgroups based on the presence of gastrointestinal involvement.

**Results:**

Among the 206 patients, 22.82% had gastrointestinal (GI+) involvement, while 77.18% had non-gastrointestinal (GI−) involvement. The GI+ group exhibited significantly higher rates of skin lesions extending to both lower limbs, atypical rashes such as blisters and necrosis, and joint involvement compared to the GI− group (*P* < 0.05). Multivariate analysis identified elevated D-dimer levels (> 1,040 μg/L) as an independent predictor for gastrointestinal involvement (OR = 1.460, 95% CI: 1.151–1.852, *P* < 0.05).

**Conclusions:**

Adult HSP patients with gastrointestinal involvement are more likely to present with extensive skin lesions, atypical rashes, and joint involvement; may be at risk of misdiagnosis; and have elevated D-dimer levels.

## 1 Introduction

Henoch–Schönlein purpura (HSP), also known as IgA vasculitis (IgAV), is defined as a small-vessel vasculitis primarily characterized by purpuric rashes, often involving the joints, gastrointestinal tract, and kidneys. Its histological hallmark is leukocytoclastic vasculitis (LCV) with IgA deposition. While HSP is more common in children (1), adult patients tend to present with more severe clinical features and a poorer prognosis (2). The pathogenic processes are driven by immune cells and inflammatory mediators and regulated by multiple factors, including genetics and the environment (3).

In adults, HSP typically presents with palpable purpura, but it is more likely to manifest with atypical skin lesions such as bullae and necrosis (4). Arthritis associated with HSP is generally transient and non-destructive, affecting over 60% of adult patients. Gastrointestinal involvement often presents with abdominal pain, diarrhea, and bleeding, with severe cases leading to intestinal obstruction, perforation, and massive gastrointestinal hemorrhage (5). Renal involvement is also more common in adults, characterized by microscopic hematuria, proteinuria, and renal insufficiency, with the risk of end-stage renal failure ranging from 11% to 27% (6, 7). Rare systemic manifestations include damage to the cardiovascular, pulmonary, nervous, and genitourinary systems (8–10). Diagnosis primarily relies on clinical features, histopathology, and direct immunofluorescence (DIF). Treatment strategies depend on disease severity: mild cases are often self-limiting, while severe cases require glucocorticoids, immunosuppressive and immunomodulatory agents, and other advanced therapies (11).

Research studies on HSP have predominantly focused on the clinical features and gastrointestinal risk factors in pediatric patients, while studies on adult patients remain relatively limited. Pediatric patients generally have a favorable prognosis, with most symptoms resolving spontaneously within 2 years. In contrast, adult patients face higher risks of gastrointestinal complications and long-term renal involvement, with recurrence rates significantly higher than in children (12). Studies indicate that systemic inflammatory markers such as neutrophil-to-lymphocyte ratio (NLR), D-dimer (DD), and fibrinogen degradation products (FDP) may help predict gastrointestinal and renal involvement (13–15). Additionally, elevated red blood cell distribution width (RDW) may be independently associated with renal damage (16). Immune dysregulation, including decreased IgG, C3, and C4 levels, is more pronounced in patients with gastrointestinal bleeding (17, 18).

This study retrospectively analyzes the clinical characteristics and laboratory parameters of adult patients with HSP to explore predictors for gastrointestinal involvement, aiming to provide a scientific basis for early identification.

## 2 Materials and methods

### 2.1 Participants

This study included patients diagnosed with HSP at the Department of Dermatology of the Second Affiliated Hospital of Xi’an Jiaotong University from May 2017 to October 2023. The diagnostic criteria consisted of an essential criterion-palpable purpura without thrombocytopenia and four secondary criteria (19): i) abdominal symptoms; ii) histopathological evidence of IgA deposition; iii) arthritis or arthralgia; and iv) proteinuria > 0.3 g/24 h, urine albumin-to-creatinine ratio > 30 mg/g, or hematuria. Patients meeting only the essential criterion were classified as having “simple HSP,” while those additionally meeting the secondary criteria were categorized as follows: “abdominal type” (criterion i), “arthritic type” (criterion iii), “renal type” (criterion iv), or “mixed type” if two or more secondary criteria were fulfilled (20).

The inclusion criteria required patients to meet the diagnostic criteria and to be ≥ 16 years of age. The exclusion criteria included a history of hematologic disorders, other autoimmune diseases, or malignancies; a history of other renal or gastrointestinal diseases; use of systemic corticosteroids or immunosuppressants within the past 30 days; or blood transfusion within the past 30 days.

Patients were divided into two groups based on gastrointestinal involvement: the gastrointestinal (GI+) involvement group and the non-gastrointestinal (GI−) involvement group. Among them, GI involvement was defined by the fulfillment of two criteria: 1) the presence of one or more of the following abdominal symptoms: abdominal pain, diarrhea, abdominal distension, nausea, vomiting, melena, hematochezia, or hematemesis (21); and 2) a positive fecal occult blood test. Patients presenting with abdominal pain, diarrhea, abdominal distension, nausea, vomiting, or occult blood in the stool were classified as having mild GI involvement. Those exhibiting melena, hematochezia, hematemesis, or ultrasound evidence of intestinal wall edema were classified as having severe GI involvement (22). The GI− group consisted of patients without any abdominal symptoms. Recurrence was defined as the reappearance of HSP-related symptoms after a symptom-free period of at least 1 month following the cessation of treatment.

### 2.2 Data collection

Study variables mainly included demographic manifestations (age, sex, and season of onset), clinical features (skin manifestations, gastrointestinal symptoms, renal involvement, joint involvement, misdiagnosis, treatment, and prognosis), laboratory examinations [white blood cell count (WBC), hemoglobin levels (HB), platelet count (PLT), neutrophil count (NEU), monocyte count (MON), lymphocyte count (LYM), RDW, NLR, platelet-to-lymphocyte ratio (PLR), lymphocyte-to-monocyte ratio (LMR), systemic immune inflammation (SII), prothrombin time (PT), activated partial thromboplastin time (APTT), fibrinogen (FIB), thrombin time (TT), DD, FDP, immunoglobulin A (IgA), immunoglobulin E (IgE), and C-reactive protein (CRP)], imaging examinations (intestinal ultrasound, upper abdominal ultrasound, or abdominal CT), and histopathology findings [hematoxylin and eosin (HE) staining and DIF].

### 2.3 Statistical analysis

Data were analyzed using SPSS 27.0. For categorical variables, data were presented as proportions and analyzed with Pearson’s chi-square, Yates’ continuity correction, or Fisher’s exact test. For continuous variables, which were expressed as mean ± standard deviation or median (interquartile range), comparisons were made using the *t*-test or the Mann–Whitney *U* test based on data distribution. Multicollinearity was assessed using variance inflation factors (VIF). Severe multicollinearity (VIF > 10) was addressed by retaining relevant variables, ensuring post-correction VIF < 5. A multivariate logistic regression model was then constructed to identify independent predictors, reporting odds ratios (ORs) with 95% confidence intervals (CIs). Multiple hypothesis tests were adjusted using the Benjamini and Hochberg false discovery rate (FDR); significant differences were considered when the results were below an FDR threshold of 0.05. Receiver operating characteristic (ROC) curves were constructed to evaluate the predictive ability of independent predictors for gastrointestinal involvement in HSP. Statistical significance was set at *α* = 0.05, with *P* < 0.05 considered statistically significant. The figure was generated using GraphPad Prism 10.1.2.

## 3 Results

### 3.1 Demographic and clinical manifestations between the GI+ and GI− groups

A total of 206 eligible HSP patients were included, with 47 patients in the GI+ group and 159 patients in the GI− group. Statistical analysis revealed no significant differences between the two groups in terms of age, season of onset, skin symptoms, or renal involvement. Conversely, these differences in age, rash distribution, atypical rashes, and joint involvement were statistically significant (**Table 1**).

**Table 1:** Demographic and clinical manifestations between the two groups.

Demographic and clinical manifestations	Gastrointestinal involvement		*z*/*χ*^2^	*P*
	GI+ (*n* = 47)	GI− (*n* = 159)		
Age, median (IQR), years	33.00 (22.00, 53.00)	37.00 (25.00, 55.00)	−0.947	0.344
Male, *n* (%)	29 (61.70)	67 (42.14)	5.580	0.018
Season of onset, *n* (%)				
Spring	12 (25.53)	38 (23.90)	0.389	0.943
Summer	12 (25.53)	48 (30.19)		
Autumn	10 (21.28)	31 (19.50)		
Winter	13 (27.66)	42 (26.41)		
Skin manifestations, *n* (%)				
Rashes extend beyond both lower extremities	27 (57.45)	39 (24.53)	18.054	<0.001
Atypical rashes other than purpura	8 (17.02)	6 (3.77)	8.068	0.005
Skin symptoms (pruritus, pain, or a burning sensation)	11 (23.40)	24 (15.09)	1.776	0.183
Renal involvement, *n* (%)	12 (25.53)	31 (19.50)	0.800	0.371
Joint involvement, *n* (%)	27 (57.45)	26 (16.35)	32.060	<0.001

IQR, interquartile range.

### 3.2 Laboratory examinations between the GI+ and GI− groups

Significant differences were observed between the two groups in WBC, PLT, NEU, MON, RDW-SD, NLR, PLR, LMR, SII, APTT, FIB, TT, DD, FDP, and CRP (**Table 2**). No significant differences were observed in the positive rates of IgA, IgM, C3, and eosinophils in the histopathology between GI+ and GI− patients (**Table 3**).

**Table 2:** Differences in hematological examinations between the two groups.

Hematological examinations	Gastrointestinal involvement		*z*/*χ*^2^	*P*
	GI+ (*n* = 47)	GI− (*n* = 159)		
WBC, 10^12^/L	10.80 (8.10, 13.17)	6.46 (5.62,8.51)	−6.834	<0.001
HB, g/L	140.00 (130.00, 160.00)	140.00 (131.00, 152.00)	−0.380	0.704
PLT, 10^9^/L	302.00 (270.00, 345.00)	241.00 (195.00, 280.00)	−4.972	<0.001
NEU, 10^9^/L	7.81 (5.82, 9.35)	3.99 (3.01, 5.39)	−7.363	<0.001
MON, 10^9^/L	0.62 (0.48, 0.78)	0.43 (0.34, 0.53)	−5.207	<0.001
LYM, 10^9^/L	2.00 ± 0.70	2.13 ± 0.77	−1.065	0.290
RDW-SD, fL	40.70 (38.60, 42.30)	42.20 (39.60, 44.90)	−2.639	0.008
RDW-CV, %	12.40 (12.00, 12.70)	12.50 (12.00, 13.10)	−0.948	0.343
NLR	3.81 (2.74, 5.54)	2.07 (1.55, 2.76)	−6.441	<0.001
PLR	152.38 (111.76, 206.08)	115.41 (90.58, 141.24)	−3.775	<0.001
LMR	3.30 (2.35, 3.89)	4.67 (3.78, 5.98)	−5.310	<0.001
SII	1,077.81 (782.96, 1,653.74)	497.68 (318.47, 697.95)	−7.051	<0.001
PT, s	10.90 (10.50, 11.30)	10.90 (10.30, 11.30)	−0.718	0.473
APTT, s	22.70 (19.80, 26.60)	25.70 (22.60, 28.70)	−3.203	0.001
FIB, mg/dL	5.46 (4.06, 358.00)	3.34 (2.59, 276.00)	−4.092	<0.001
TT, s	17.10 (15.90,17.90)	18.00 (17.00, 19.00)	−4.342	<0.001
DD, μg/L	3,950.00 (1,560.00, 6,170.00)	560.00 (400.00, 980.00)	−8.414	<0.001
FDP, mg/L	9.28 (5.38, 14.26)	1.69 (1.14, 3.24)	−8.535	<0.001
Serum IgA > ULN, *n* (%)	10/36 (27.78)	17/70 (24.29)	1.153	0.696
Serum IgE > ULN, *n* (%)	15/36 (41.67)	21/70 (30.00)	1.443	0.230
CRP > ULN, *n* (%)	19/24 (79.17)	20/50 (40.00)	9.980	0.002

GI, gastrointestinal involvement; WBC, white blood cell count; HB, hemoglobin levels; PLT, platelet count; NEU, neutrophil count; MON, monocyte count; LYM, lymphocyte count; RDW, red blood cell distribution width; NLR, neutrophil-to-lymphocyte ratio; PLR, platelet-to-lymphocyte ratio; LMR, lymphocyte-to-monocyte ratio; SII, systemic immune inflammation; PT, prothrombin time; APTT, activated partial thromboplastin time; FIB, fibrinogen; TT, thrombin time; DD, D-dimer; FDP, fibrinogen degradation products; IgA, immunoglobulin A; ULN, upper limit of normal; IgE, immunoglobulin E; CRP, C-reactive protein.

**Table 3:** Differences in histopathological examinations between the two groups.

Histopathological examination	Gastrointestinal involvement		*χ* ^2^	*P*
	GI+ (*n* = 47)	GI− (*n* = 159)		
IgA+, *n* (%)	9/11 (81.82)	13/24 (54.17)		0.150
IgM+, *n* (%)	0/11 (0.00)	4/24 (16.67)		0.285
C3+, *n* (%)	6/11 (54.55)	12/24 (50.00)		1.000
EOS infiltration, *n* (%)	4/13 (30.77)	13/29 (44.83)	0.736	0.391

GI, gastrointestinal involvement; IgA, immunoglobulin A; IgM, immunoglobulin M; C3, complement 3; EOS, eosinophil.

### 3.3 Characteristics of patients with gastrointestinal involvement

The main GI manifestations are detailed in **Table 4**. All GI+ patients experienced abdominal pain, and some exhibited multiple gastrointestinal symptoms simultaneously. For GI+ patients, 16 cases (34.04%) presented with purpura and abdominal pain as initial symptoms. 4 cases (8.51%) initially presented with abdominal pain alone, in which 2 (50.00%) were misdiagnosed. Delayed onset of abdominal pain occurred up to 25 days after the appearance of purpura, with a median delay of 6.00 (1.25, 11.00) days.

**Table 4:** Characteristics of patients with gastrointestinal involvement.

Characteristics	*n* (%)
Clinical characteristics	47
Abdominal pain	47 (100.00)
Diarrhea	15 (31.91)
Nausea and vomiting	7 (14.89)
Melena	7 (14.89)
Hematochezia	5 (10.64)
Abdominal distension	2 (4.26)
Hematemesis	1 (2.13)
Fecal routine examination	47
Positive fecal occult blood test	47 (100.00)
Radiological features (intestinal ultrasound or CT imaging)	41
Normal	33 (80.49)
Edema and thickening of bowel wall	8 (19.51)
Diagnosis	47
Diagnosed with HSP	36 (76.60)
Diagnosed with other diseases	11 (23.40)
Acute gastroenteritis	4 (8.51)
Gastrointestinal ulcer	2 (4.26)
Appendicitis	1 (2.13)
Urticaria	1 (2.13)
Unexplained abdominal pain	1 (2.13)
Erythema multiforme	1 (2.13)
Allergic dermatitis	1 (2.13)

### 3.4 Treatment

Of 201 patients with treatment regimens, 105 patients had the simple type involving only skin lesions. Among these, 34 patients (32.38%) received corticosteroid treatment, while 71 (67.62%) did not. A total of 96 patients had joint, gastrointestinal, and/or renal involvement, of whom 90 (93.75%) received corticosteroid treatment, and 6 (6.25%) did not. For patients with the simple type, the average methylprednisolone dosage was 0.59 ± 0.18 mg/kg/day. For those with joint, gastrointestinal, and/or renal involvement, the average methylprednisolone dosage was 0.73 ± 0.22 mg/kg/day. Additionally, 1 patient with abdominal-type HSP combined with renal and joint involvement received a combination treatment of dexamethasone and cyclophosphamide, while another with abdominal-type HSP and renal involvement underwent a triple therapy regimen of dexamethasone, cyclophosphamide, and immunoglobulin.

### 3.5 Prognosis

No significant difference was observed in the duration of illness between the two groups. However, the length of hospitalization was significantly longer in the GI+ group. 35 patients experienced recurrence, with all patients having one recurrence. The shortest time to recurrence was 1 month, and the longest was 10 years, with a median time to recurrence of 13.50 (4.00, 42.00) months. The recurrence rates did not show a significant difference (**Table 5**).

**Table 5:** Differences in prognosis between the two groups.

Prognosis	Gastrointestinal involvement		*z*/*χ*^2^	*P*
	GI+ (*n* = 47)	GI− (*n* = 159)		
Duration, median (IQR), days				
Duration of illness	10.00 (7.00, 15.00)	14.50 (6.00, 30.00)	−0.281	0.779
Duration of hospitalization	7.00 (6.00, 10.00)	6.00 (5.00, 7.00)	−3.941	<0.001
Recurrence, *n* (%)	6 (12.77)	29 (18.24)	0.770	0.380

GI, gastrointestinal involvement; IQR, interquartile range.

### 3.6 Predictors for gastrointestinal involvement in HSP

Univariate analysis between the GI+ and GI− groups identified several indicators with statistically significant differences and complete data. Multicollinearity analysis of these indicators revealed significant collinearity between WBC, NEU, NLR, PLR, SII, DD, and FDP (VIF > 10), as shown in **Table 6**. Since WBC and NLR primarily increase due to changes in NEU, and SII = PLR × NEU, with DD being a major component of FDP, further analysis excluding WBC, NLR, SII, and FDP showed that the VIF for the remaining indicators was all < 5, as shown in **Table 6**. Therefore, rash distribution, atypical rashes, PLT, NEU, MON, PLR, LMR, APTT, FIB, TT, and DD were included in a multivariate binary logistic regression model. The results indicated that elevated DD could be an independent predictor for gastrointestinal involvement in HSP (**Table 7**).

**Table 6:** Collinearity analysis of related factors for gastrointestinal involvement in HSP.

Related factors	Collinearity analysis			Corrected collinearity analysis		
	Tolerance	VIF	*P*	Tolerance	VIF	*P*
Age	0.910	1.099	0.268	0.920	1.087	0.213
Rash distribution	0.798	1.253	0.014	0.810	1.234	0.028
Atypical rashes	0.754	1.326	0.772	0.824	1.213	0.805
WBC	0.008	118.640	0.003			
PLT	0.146	6.866	0.033	0.480	2.084	0.762
NEU	0.009	109.640	0.004	0.419	2.386	0.002
MON	0.122	8.204	0.008	0.299	3.342	0.634
NLR	0.015	66.412	0.303			
PLR	0.075	13.387	0.056	0.374	2.671	0.715
LMR	0.196	5.097	0.058	0.366	2.734	0.903
SII	0.031	32.367	0.578			
APTT	0.781	1.281	0.108	0.837	1.195	0.257
FIB	0.730	1.369	0.084	0.800	1.250	0.085
TT	0.753	1.328	0.009	0.764	1.308	0.003
DD	0.017	59.555	0.804	0.725	1.380	<0.001
FDP	0.016	60.674	0.693			

WBC, white blood cell count; PLT, platelet count; NEU, neutrophil count; MON, monocyte count; NLR, neutrophil-to-lymphocyte ratio; PLR, platelet-to-lymphocyte ratio; LMR, lymphocyte-to-monocyte ratio; SII, systemic immune inflammation; APTT, activated partial thromboplastin time; FIB, fibrinogen; TT, thrombin time; DD, D-dimer; FDP, fibrinogen degradation products.

**Table 7:** Logistic regression analysis for gastrointestinal involvement in HSP.

Related factors	*B*	Wald	*P*	Corrected *P*	OR	95% CI	
						Lower limit	Upper limit
Age	0.659	1.628	0.202	0.348	1.932	0.703	5.315
Rash distribution	−0.762	1.909	0.167	0.348	0.467	0.158	1.375
Atypical rashes	−0.256	0.099	0.753	0.822	0.774	0.157	3.806
PLT	0.006	1.622	0.203	0.348	1.006	0.997	1.015
NEU	0.195	2.825	0.093	0.279	1.215	0.968	1.525
MON	0.085	0.172	0.678	0.822	1.089	0.729	1.626
PLR	0.002	0.100	0.752	0.822	1.002	0.991	1.013
LMR	0.050	0.043	0.836	0.836	1.051	0.653	1.692
APTT	−0.054	0.803	0.370	0.555	0.948	0.843	1.066
FIB	0.003	4.678	0.031	0.124	1.003	1.000	1.006
TT	−0.523	6.189	0.013	0.078	0.593	0.393	0.895
DD	0.378	9.703	0.002	0.024	1.460	1.151	1.852

PLT, platelet count; NEU, neutrophil count; MON, monocyte count; PLR, platelet-to-lymphocyte ratio; LMR, lymphocyte-to-monocyte ratio; APTT, activated partial thromboplastin time; FIB, fibrinogen; TT, thrombin time; DD, D-dimer.

**Figure 1:**
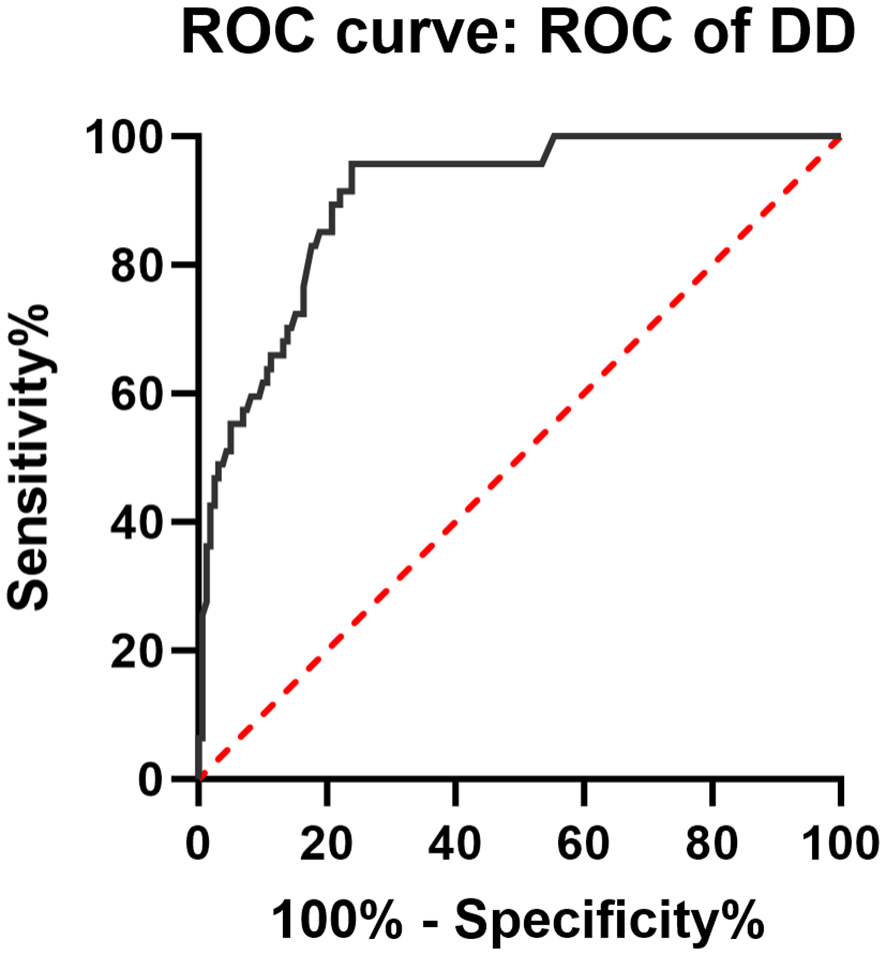
ROC curves for predicting gastrointestinal involvement in HSP.

The multivariate logistic regression analysis identified statistically significant indicators, which were used to construct ROC curves. The area under the curve (AUC) for DD in predicting gastrointestinal involvement in HSP was 0.904 (95% CI: 0.860–0.948, *P* < 0.001) (**Figure 1**). When DD > 1,040 μg/L, the risk of gastrointestinal involvement in HSP increased. The sensitivity for predicting gastrointestinal involvement using DD was 95.70%, and the specificity was 76.10%. When comparing the DD levels of patients with mild and severe gastrointestinal involvement, the results showed that patients with severe gastrointestinal involvement had higher DD levels; however, this difference was not statistically significant (*P* = 0.067).

## 4 Discussion

We focused on the comparison of clinical characteristics and laboratory results in adult HSP patients between the GI+ group and the GI− group. Among 206 adult HSP patients, those in the GI+ group were more likely to have widespread skin lesions. A study from Beijing Children’s Hospital also suggested that widespread rashes are associated with the severity of gastrointestinal bleeding, particularly facial rashes, which could serve as an early warning signal for gastrointestinal hemorrhage (23). GI+ patients were more likely to have atypical rashes compared to those in the GI− group. Previous studies on the association between atypical skin lesions and organ involvement have been inconsistent. Belli et al. found that bullous or necrotic lesions were associated with gastrointestinal and renal involvement, while Tancrede-Bohin et al. did not observe such a correlation (24).

Among 47 patients from the GI+ group in this study, abdominal pain was the most common symptom, followed by diarrhea, nausea, and vomiting. These symptoms are likely related to edema and hemorrhage of the gastrointestinal wall and mesentery, consistent with the findings from a study in Korea (25). Although previous research suggested that severe gastrointestinal involvement in pediatric IgAV might be significantly associated with renal involvement (21, 22), this study, consistent with a French study, did not find a significant difference in the degree of renal involvement in adults with HSP in the GI+ group (26). In terms of joint involvement, 53 patients predominantly had knee and ankle involvement, suggesting that HSP-associated arthritis tends to affect larger joints, consistent with prior literature (26). The proportion of joint involvement was significantly higher in the GI+ group, further emphasizing the potential association between gastrointestinal and joint symptoms.

In children with HSP, the interval between abdominal pain and rash is typically 2–10 days, with the longest reported interval of 28 days in untreated patients (27, 28). Moreover, previous studies have reported that 14%–36% of patients experience gastrointestinal symptoms prior to the onset of purpura, which are significant risk factors for conditions such as intestinal intussusception and perforation (29, 30). In our study, adult patients usually experienced abdominal pain after the onset of rashes, with a median delay of 6 days and a maximum delay of up to 30 days. Among the GI+ patients, 16 cases (34.04%) presented with purpura accompanied by abdominal pain as their initial symptom, while 4 cases (8.51%) had abdominal pain as the sole presenting symptom. The delay and disguise of gastrointestinal symptoms make diagnosis and treatment more challenging. The result is that 20 patients were diagnosed with other diseases among 206 adult HSP patients. The misdiagnosis rate was 9.71%, with a higher rate in the GI+ group (23.40%). In patients with abdominal pain as the sole presenting symptom, the misdiagnosis rate was as high as 50.00%. These misdiagnosed patients were often treated with routine gastric mucosal protection therapy, potentially resulting in serious complications such as intestinal necrosis and perforation. Previous studies have also mentioned that some patients underwent unnecessary surgeries due to misdiagnosis (31). Therefore, identifying precise and cost-effective biomarkers to predict gastrointestinal involvement is crucial.

Multivariate logistic regression analysis, combined with ROC curves, confirmed that a DD level > 1,040 µg/L was an independent predictor for gastrointestinal involvement in adults. The mechanism may be closely related to the pathological process of HSP. Abnormal glycosylation of IgA1 (Gd-IgA1) leads to the formation of immune complexes that deposit on the walls of small blood vessels throughout the body, triggering sterile small-vessel inflammation (3). This vasculitis further damages the endothelial structure, exposing subendothelial collagen and activating the coagulation pathway (32). Meanwhile, the microthrombi formed by platelet activation trigger compensatory fibrinolysis, during which plasmin degrades cross-linked fibrin to produce DD, ultimately resulting in elevated DD levels (33). This finding is consistent with previous studies that observed an association between elevated DD levels and gastrointestinal and other systemic involvements in IgAV patients (23, 34, 35).

This study did not find a direct link between high IgA levels and gastrointestinal involvement in adult patients, which is in line with prior research (26). Although IgE levels were elevated in some patients, no significant relationship was found between IgE levels and gastrointestinal involvement. Previous studies have confirmed that serum IgE elevation is common in children with HSP, with higher IgE levels in children with gastrointestinal bleeding compared to those with non-gastrointestinal bleeding (36). Meanwhile, most HSP patients present with an elevated number of Th2 and Th17 cells (3). IL-4 drives the conversion of B cells into plasma cells, leading to the production of IgE (37, 38). Additionally, studies have suggested that children with Th2-driven allergic conditions like allergic conjunctivitis, allergic rhinitis, and atopic dermatitis are at a higher risk for developing HSP and HSP nephritis (39). These studies prove that HSP may involve multiple immune pathways, including changes in Th1/Th2 balance and IgE-mediated mechanisms.

CRP levels were significantly elevated in the GI+ group. As an acute-phase reactant, CRP directly activates endothelial cells, promoting the release of inflammatory mediators and adhesion molecules, which indirectly activate the immune system and lead to the activation of inflammatory cells (40). These processes contribute to the development of IgAV by initiating and amplifying the inflammatory response within the blood vessel walls (41). This suggests a potential association between inflammation levels and gastrointestinal involvement. However, the relationship between CRP and renal involvement in HSP remains controversial (26, 41).

In terms of prognosis, HSP patients in the GI+ group had a significantly longer hospital stay, but the duration of illness did not increase significantly. This is related to the individualized treatment plans that patients received in this retrospective study. Just as a French study indicated that the prognosis of patients with or without gastrointestinal involvement was similar (26), there was also a difference in treatment regimens that masked the true negative impact of gastrointestinal involvement on prognosis (42). The recurrence rate of HSP varies widely across studies, ranging from 2.7% to 51.7% (43). In this study, the recurrence rate was 16.99%, though it may be underestimated due to patient dropout. While it is traditionally believed that relapses typically occur within 6 months of the initial onset, the median time to recurrence in this study was 13.50 months, with the longest recurrence occurring after 10 years. Recurrences varied in presentation but were generally similar to the initial disease manifestation. In this study, all relapsed patients presented with skin rashes only, although the possibility of underlying renal involvement was not excluded. Previous studies have shown that widespread or necrotic skin lesions may increase the risk of relapse in children (44), and adult HSP patients with gastrointestinal involvement are at a higher risk for recurrence (45). However, no such associations were found in this study. We found that patients with relapse had a longer duration of illness, which may be related to insufficient initial treatment, suggesting that “inflammatory memory” could be a potential risk factor for recurrence.

However, our study also has several limitations. First, the sample size is limited, especially with a small number of biopsy-confirmed cases. Moreover, the inherent bias of retrospective studies and inaccurate recurrence data due to unclear recall need to be interpreted with caution. Additionally, the single-center design may introduce selection bias. Future multicenter and prospective studies are needed to validate these findings.

## 5 Conclusion

Adult HSP patients in the GI+ group are more likely to exhibit rashes extending beyond the lower limbs, atypical rashes (such as bullae and necrosis), and joint involvement. Elevated DD levels may serve as an independent predictor for gastrointestinal involvement in HSP, while renal involvement and DIF results did not show a significant association with gastrointestinal involvement. Additionally, patients with gastrointestinal involvement may be at risk of misdiagnosis as other digestive system disorders.

## Data Availability

The raw data supporting the conclusions of this article will be made available by the authors, without undue reservation.
